# Visualization of dental plaque with a 3D-intraoral-scanner—A tool for whole mouth planimetry

**DOI:** 10.1371/journal.pone.0276686

**Published:** 2022-10-26

**Authors:** Katja Jung, Katja Giese-Kraft, Melanie Fischer, Kai Schulze, Nadine Schlueter, Carolina Ganss

**Affiliations:** 1 Department of Conservative and Preventive Dentistry, Dental Clinic of the Justus-Liebig-University Giessen, Giessen, Germany; 2 M-Pro, Graz, Austria; 3 Department of Conservative Dentistry, Periodontology and Preventive Dentistry, Hannover Medical School, Hannover, Germany; Centre Hospitalier Regional Universitaire de Tours, FRANCE

## Abstract

Planimetry is a reliable method for detecting and monitoring plaque. Until now, this method has mainly been applied to conventional-camera images, which is difficult and time-consuming in relation to the entire dentition. Today, 3D-intraoral-scans are well suited for imaging the entire dentition and are therefore an efficient and feasible alternative. 3D-intraoral-scans have already proven successful for the quantification of plaque based on a plaque index. Therefore, aim of this study was to investigate whether images from 3D-intraoral-scans are also suitable for valid planimetric plaque measurements and monitoring; intraoral-camera images served as a reference. Twenty subjects (27.5±1.2 years) were included. Plaque was disclosed at three different time points: habitual plaque (T1), after 72 h without oral hygiene (T2) and after subsequent tooth brushing (T3) and quantified using 3D-intraoral-scans and intraoral-camera images (intraoral-camera CS 1500, intraoral-scanner CS 3600; Carestream Dental, Germany). The percentage of the plaque-covered surface of the total surface area (P%) was determined with a software specially programmed for this purpose using images from 3D-intraoral-scans of the oral and vestibular surfaces of the Ramfjord teeth (16, 21, 24, 36, 41, and 44); the intraoral-camera images of the vestibular surfaces of 16 and 36 served as reference. P% from images of the 3D-intraoral-scan and the intraoral-camera revealed a very good correlation (r = 0.876; p ≤ 0.001); the Bland-Altmann analysis showed a good agreement with no proportional and a very minor systematic bias with slightly higher values from images of the 3D-intraoral-scan. Further, P% measurements of the images of the 3D-intraoral-scan were able to detect changes in plaque levels, showing a 47% (p ≤ 0.001) increase in P% from T1 to T2 and a 43% (p ≤ 0.001) decrease after toothbrushing (T3). Planimetry using images of the 3D-intraoral-scan seems to be a suitable tool for whole mouth planimetry to record and monitor dental plaque.

## Introduction

Traditionally, plaque levels are recorded chairside using index systems, which code amounts of plaque in numbers [1]. This procedure has the obvious disadvantage that the clinical findings are not recorded in images and the reliability of the survey depends on the skill of the examiner. Furthermore, depending on the number of examiners and the complexity of the used index, more or less extensive calibrations are required, and recoding into other indices at a later stage is only possible to a limited extent. The main disadvantage of the different clinical indices is that they are not comparable with each other and potentially provide different results for the same question. An example for this is that the effect size of plaque reduction in toothbrush studies depends largely on the plaque index used. Respectively, systematic reviews evaluating different types of toothbrushes found plaque score reductions of 52% with the Navy index as compared to 30% with the Q&H index for manual toothbrushes [2] and of 65% and 36%, respectively, for powered brushes [3]. This problem is well known and has been nicely discussed in a recent systematic review on the effectiveness of high-frequency sonic versus oscillating-rotating powered toothbrushes [4]. In addition, it is known that not every plaque index is equally suitable for every question and should be adapted accordingly [5]. A solution to this could be plaque planimetry using 3D-intraoral-scans, which can objectively quantify the amount of plaque and permanently keep a record of the oral hygiene status.

Planimetry has been used with many variations to analyse conventional or fluorescence images [6–14] but it is still not very well established. One of the main reasons for this could be that capturing intraoral photos is very time-consuming and technically demanding, which makes the technique more suitable for special questions, for the assessment of individual teeth or for smaller examination groups.

However, the new digital intraoral imaging techniques could make planimetry available for much broader applications. In particular, intraoral-scanners can digitise the entire dentition in a short time, which could solve the problem of technically demanding intraoral photography.

A recent publication gives the first indications that 3D-intraoral-scanners may be suitable for plaque detection [15]. However, only eight patients were included in this study with only one examination time point.

The objective of the present study was therefore to investigate whether plaque can be validly detected and monitored using images from the 3D-intraoral-scan. For this, plaque amounts were measured planimetrically at three different time points: habitual plaque, plaque after 72 hours without oral hygiene, and after subsequent tooth brushing.

## Subjects, materials and methods

The study was a prospective study in which dental plaque was evaluated planimetrically using images from a 3D-intraoral-scan. Intraoral-camera images were used as a reference method for this purpose. Both the 3D-intraoral-scans and the intraoral-camera images are used for this purpose were obtained in conjunction with a previously published study [16].

The study was approved by the Ethics Committee of the Justus Liebig University Giessen (Doc. No. 142/19). Informed consent was obtained in written form. It took place in the Department of Conservative and Preventive Dentistry of the Dental Clinic of the Justus Liebig University Giessen. It complied with the Declaration of Helsinki and the guidelines of Good Clinical Practice [17].

### Study procedure

The clinical procedure for imaging collection has been extensively outlined earlier [16]. Therefore, it is only briefly described again here.

**Fig 1:**
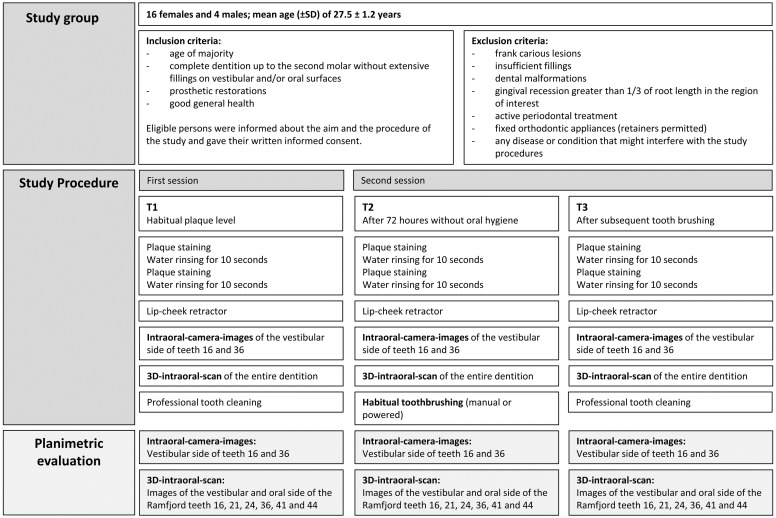
Flow chart of study procedures.

The inclusion and exclusion criteria as well as the study procedure is shown in Fig 1.

We examined the plaque amount in each participant at three different time points.

First, a habitual plaque level (T1), second, after 72 hours without oral hygiene, and third, immediately after tooth brushing (T3).

For all three observation times (T1 –T3) dental plaque was disclosed with Mira-2-Ton^®^ solution (Hager & Werken GmbH & Co Kg, Duisburg, Germany). The disclosing agent was applied with a saturated foam pellet. This disclosing procedure was repeated twice and in between, the mouth was rinsed with water for 10 seconds. Afterwards, a lip-cheek retractor (OptraGate; Ivoclar Vivadent, Schaan, Liechtenstein) was inserted and the area of interest was kept dry by sucking off saliva and using an air blower. At first, the buccal surface of the upper right and the lower left molars (teeth 16 and 36) was captured with an intraoral-camera (intraoral-camera CS1500 Carestream Dental LLC, Atlanta, United States and Stuttgart, Germany). The intraoral camera head has white LED lights arranged in a circle around the lens window to ensure good illumination of the area to be imaged, even in darker areas such as the posterior molar region.

This illumination is sufficient for normal use. In our case, we additionally illuminated the area of interest with the dental light to achieve optimum contrast between the tooth surface and the plaque areas. Following the camera images the entire dentition was scanned with a 3D-intraoral-scanner (intraoral-scanner CS 3600 Carestream Dental LLC, Atlanta, United States and Stuttgart, Germany). In contrast to the exposures with the intraoral camera, the dental light was switched off during the scanning process. The additional light source leads to light reflections on the tooth surface, especially in the anterior region, which results in an undesirable reduction of the scan quality. The illumination by the scanner itself led to a homogeneous illumination of the scan area.

For T3, the participants brushed their teeth right after collecting data for T2, in the same session. Each participant could choose the type of toothbrush depending on their habit, either a manual toothbrush (elmex^®^ sensitive, GABA GmbH, Swiss, Colgate Palmolive Company, USA) or a powered toothbrush (Professional Care 3000 oral-B^®^, powered by BRAUN GmbH, Germany, Procter & Gamble, USA).

At the end of each session (T1 and T3), after all data has been collected, a professional tooth cleaning was performed, using polishing paste and a rubber cup, to ensure that the disclosing agent was completely removed and the dentition was free of plaque accumulation.

After image acquisition, a planimetric analysis of the images from the 3D-intraoral-scans and the intraoral-camera was performed. For this purpose, the percentage of the plaque-covered surface of the total surface area (P%) at all three time points (T1-T3) was determined using a software specially programmed for this purpose. Planimetric analysis was performed for images from the 3D-intraoral-scans of Ramfjord teeth (16, 21, 24, 36, 41 and 44) from both the oral and vestibular tooth surfaces and for the intraoral-camera images of the vestibular surface of teeth 16 and 36.

While the centrally aligned intraoral-camera image, in which both the mesial and distal parts could be seen equally well, could be used directly for the evaluation an image of the tooth in question was first required for the evaluation of the 3D-intraoral-scan.

#### Creation of an image from a 3D-intraoral-scan

The 3D-intraoral-scans were saved as an STL-file and could be called up again in the CS Mesh Viewer (Carestream Dental, Germany). In this viewer, the 3D-intraoral-scan can be rotated in all three directions (x, y and z-axis) so that all areas of the dental arch can be well displayed.

Images of the 3D-intraoral-scan were required for our plaque evaluation. For this purpose, screenshots of the 3D-intraoral-scans of the teeth of interest were made from the vestibular as well as from the oral surfaces. All screenshots were taken at full screen size to avoid distortion due to inconsistent image sizes, and saved as JPG images.

Extensive pretests have shown that these images of the 3D-intraoral-scan had to be standardized because deviations by tilts in the x-axes and the y-axes, rotations in the z-axes and in particular the zoom factor had a decisive impact on P%.

To ensure that all participants’ baseline images are aligned in the same way, the following standards were defined. The alignment criteria of the scan to create a screenshot differed significantly between the vestibular and oral surfaces.

For the images of the vestibular sides, the first step was to enlarge the intraoral-3D-scan so that the first respectively third quadrants (for zoom settings of the maxilla and mandible, respectively) could be seen in full size on a 15.6-inch display. The scan was aligned to provide an orthograde view of tooth 16 or 36, respectively, and the mesial and distal portions of the vestibular surfaces of the corresponding tooth were equally visible.

If the orientation of the scan were to be to a different tooth when the zoom setting was adjusted, this would result in a rotation of the scan, which would change the view of the scan and thereby also change the zoom setting when adjusting to the full screen size. For this reason, the zoom setting for the vestibular surfaces was defined once by aligning to tooth 16 or 36 and was retained for teeth 21 and 24 or 41 and 44.

In the second step, the scan was aligned with the occlusal plane to minimize deviations in the x and y directions. For teeth 21 and 24 or 41 and 44, respectively, the scan was rotated in the z-direction until an orthograde view of the tooth under examination was possible. Care was taken to ensure that both the mesial and distal portions of the vestibular surface were equally visible. If necessary, the scan was realigned to the occlusal plane. The zoom settings remained unchanged as described above.

These settings could not be adopted for the oral surfaces due to the anatomy of the jaws. The contralateral side of the jaw often obscures the oral surfaces by rotating the z-axis alone. Instead, for each tooth of interest, a section of the dental arch with a suitable number of adjacent teeth (upper right first molar: area 14–17; upper left incisor: area 13–23; upper left premolar: area 22–25; lower left first molar: area 34–37; lower right first incisor: area 34–44; lower right first premolar: area 42–46) was chosen to standardize the zoom setting. This section was enlarged to full size. The inclination in the y-direction of the premolar and molar region was adjusted in a way that the vestibular cusps were at the same level as the oral cusps. Afterwards the z-direction was modified until the mesial and distal parts of the oral surface were equally visible.

In the anterior region, the incisal edge of the tooth of interest was aligned with the lower edge of the screen, taking care to display the oral surface as best as possible.

**Fig 2:**
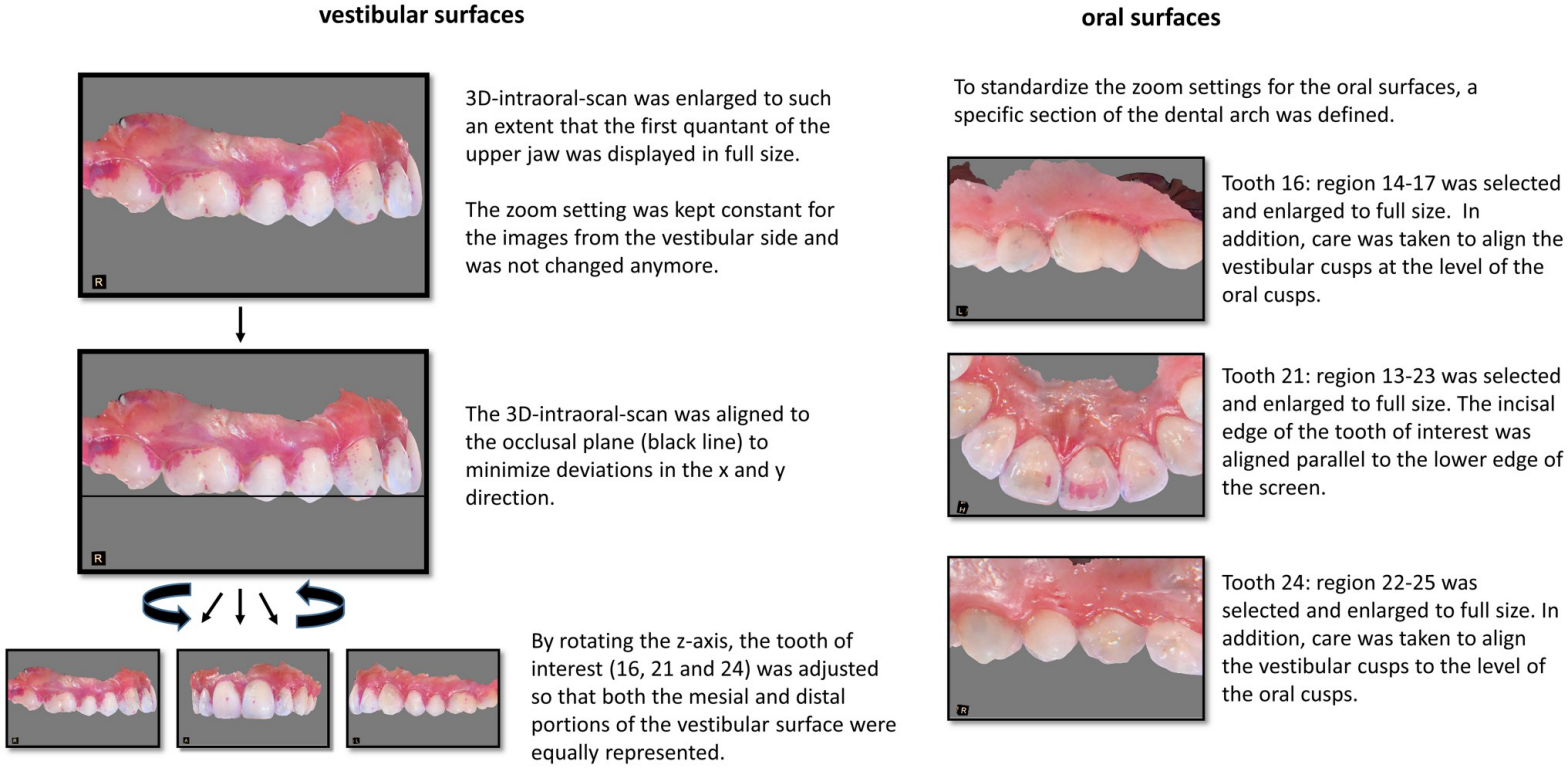
Illustration of scan alignment using the example of an upper jaw; lower jaw scans were processed accordingly.

This setting procedure is illustrated in Fig 2.

To avoid deviations from T1 to T3 of a participant, the 3D-intraoral-scan from T1 was used to create a base image of each Ramfjord tooth, which served as a template for T2 and T3.

#### Planimetric plaque measurement

The images of the intraoral-camera and those created from the 3D-intraoral-scan were imported in an image programme (Photoshop CS5 extended, Version 12.0 x 64) and the tooth of interest was cut and placed on a black background on a pen display (Cintiq 16 Display with a Wacom Pro Pen 2, Wacom Europe GmbH, Duesseldorf, Germany). To identify pixels related to plaque-covered areas, a software specially programmed for this purpose was written by using the programming language Julia (version 1.6.28) [18] and the packages “ImageMagick” [19] and “Images” [20]. The program determines the individual RGB colour components from an image saved in JPG format. The red, green and blue colour values of each pixel were determined, transformed into a 3-component vector system and stored separately. Two filters were used to calculate the plaque level. The first filter removed all black pixels of the black background (RGB = 0,0,0). The second filter was used to quantify the amount of plaque and could be adjusted individually.

**Fig 3:**
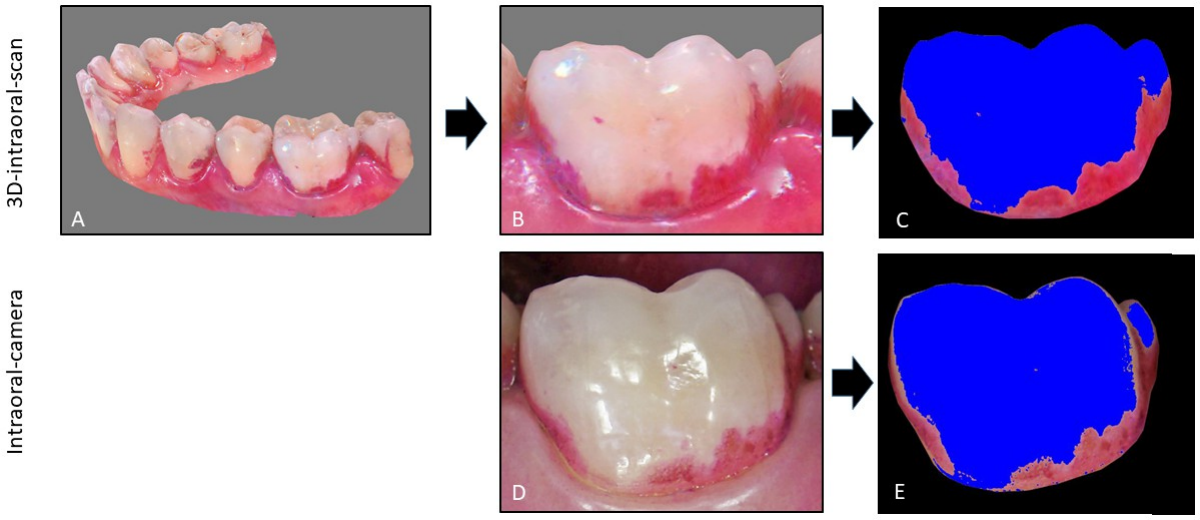
Procedure of planimetric evaluation on a 3D-intraoral-scan and an intraoral-camera image. A, B and C show the procedure for a 3D-intraoral-scan. A: the 3D-intraoral-scan itself, B: image of the tooth of interest from a 3D-intraoral-scan, C: Visualisation of the plaque amount by converting all pixels below a certain threshold value for red by the programme on a cropped tooth on a black background. D and E shows the procedure for an intraoral-camera image. D: intraoral-camera image itself, E: Visualisation of the plaque amount by converting all pixels below a certain threshold value for red by the programme on a cropped tooth on a black background.

Since each coloured pixel (all pixels except black) contains a certain amount of red, it was necessary to set a threshold above which the red component represents red-stained plaque. To define this threshold, we first set the threshold for the green and blue components to zero and adjusted the red component until the demarcation of pixels in plaque-covered and non-plaque-covered areas corresponded to the visual impression (Fig 3). Visual inspection of the threshold was performed directly in the program. All pixels with a lower red content were coloured blue. This allowed visual discrimination between areas defined as "plaque" and those defined as "plaque-free".

Fig 3. shows that the plaque area identified by the program corresponds with the visual impression of the plaque area on the original images; plaque-free areas identified by the program are coloured in blue.

For the 3D-intraoral-scan images, where the illumination by the scanner light was the same for all teeth, the same settings could be used for all images and batch processing could be performed.

However, for the intraoral camera images, the two light sources, the LED lights around the camera lens and the light from the dental unit, were not sufficient to achieve homogeneous illumination of all teeth. As a result, batch processing of the images was not possible and the threshold had to be adjusted for each image individually until the identified plaque area conformed with the disclosed plaque area on the tooth.

#### Statistics

Statistics were done with IBM SPSS Statistics version 25 (IBM Germany GmbH, Ehningen, Germany). Plaque amounts were quantified as the percentage of pixels defined as plaque-covered of the total number of pixels (P%). Plaque amount on intraoral-camera images is expressed in P%C, and the plaque amount on images from a 3D-intraoral-scan is expressed in P%S.

Kolmogorov-Smirnov-tests revealed significant differences from the Gaussian distribution for many variables; therefore, non-parametric procedures were generally used.

In order to test the reproducibility of the entire planimetric procedure, one of the 20 participants in the study was drawn at random using a random number generator. The 3D-intraoral-scan of this subject was evaluated in a blinded way a second time at intervals of about 2 weeks after the plaque measurements were completed. The evaluation included the alignment of the 3D-intraoral-scans and the creation of the images of the 3D-intraoral-scans, the cropping of the teeth and the automated plaque analysis. The median of the difference between these two evaluations P%S1 and P%S2 and 95% confidence intervals after bootstrapping (method of sampling: simple, number of samples: 2000) as well as intra class coefficient (ICC) estimates and their 95% confident intervals (CI) were calculated based on a single rating, absolute-agreement, 2-way mixed-effects model.

Bland-Altman-procedures [21, 22] were used for determining the agreement of plaque amounts obtained from the images of the 3D-intraoral-scan with values from the images of the intraoral-camera as the reference. To this end, the differences between the two methods were plotted against their mean values. Since the differences of the two methods were not normally distributed, these data were logarithmised; afterwards the Kolmogorov-Smirnov-test showed no significant deviation from the normal distribution. A regression analysis was performed to evaluate whether the order of values has an effect on agreement (proportional difference) and a one-sample t-test of the mean differences of values obtained from the two methods was done to evaluate whether there is a systematic bias. In addition, Spearman’s rank correlation was computed to assess the relationship between P%S and P%C.

The plaque amounts were analysed at the subject level by determining the total plaque amount of each subject as the mean value of the plaque values of all tooth surfaces. This was done separately for the vestibular and oral surfaces. Comparisons between T1 and T2 as well as between T2 and T3 were done with the Wilcoxon-test, the level of significance was set at .008 (Bonferroni correction). In a further step, the plaque values at the site level were considered and the differences between T1 and T2 as well as between T2 and T3 were also analysed with the Wilcoxon-test. The level of significance was set at .002 (Bonferroni correction).

## Results

### Reproducibility

The median difference (95% confidence interval) between P%S1—P%S2 was 0.06 (0.14;0.13), the maximum was 1.97 and the minimum was -5.35. The ICC (95% confidence interval) was 0.994 (0.988;0.992; p ≤0.001) indicating an excellent reproducibility [23].

### Agreement of amounts of plaque obtained from images of the 3D-intraoral-scan and the images of the intraoral-camera

**Fig 4:**
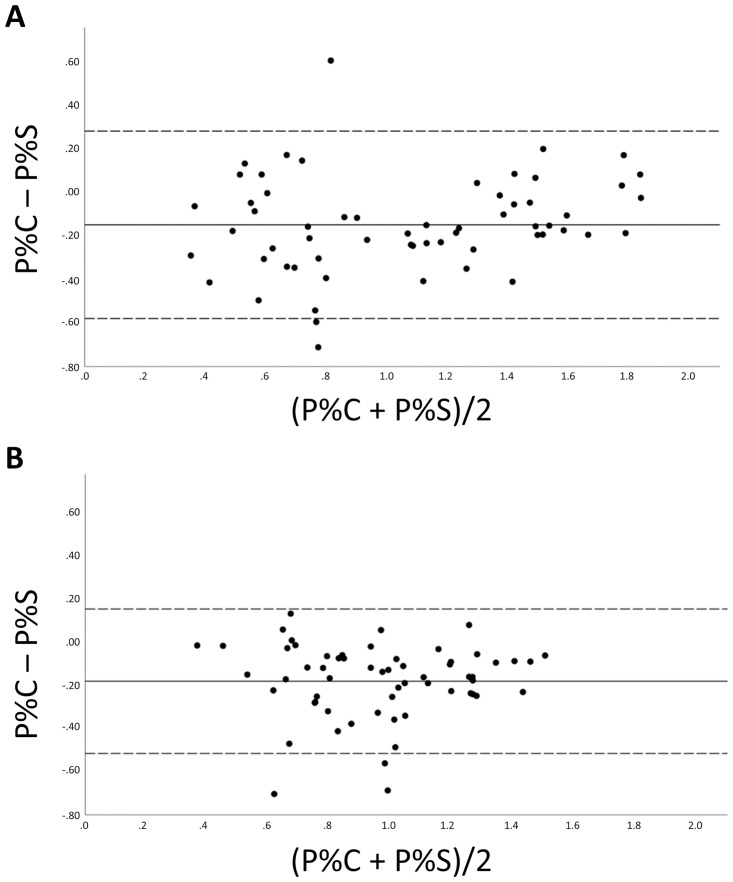
Bland-Altman plots evaluating the agreement between the plaque amounts obtained from images of the 3D-intraoral-scan with the plaque amounts obtained from images of the intraoral-camera; P%S and P%C after logarithmic transformation. A: upper right molar, B: lower left molar. The solid line indicates the mean difference of the methods of comparison; the broken lines indicate the 95% limits of agreement (mean±1.96xsd).

The Bland-Altmann analyses (Fig 4) revealed a small systematic bias as slightly higher P% values were found on images of the 3D-intraoral-scan compared to images from the intraoral-camera; the median was -3.9 (-5.8;-2.2; p≤0.001) for the upper right molar and -3.8 (-5.4;2.6; p≤0.001) for the lower left molar. The regression analysis showed no significant proportional bias (upper right molar: p = 0.127); (lower left molar: p = 0.902). The correlation coefficients for P%C and P%S were 0.876 (p≤0.001) for the upper right and 0.807 (p≤0.001) for the lower left molar.

### Plaque amounts

#### Analysis at the subject level

**Fig 5:**
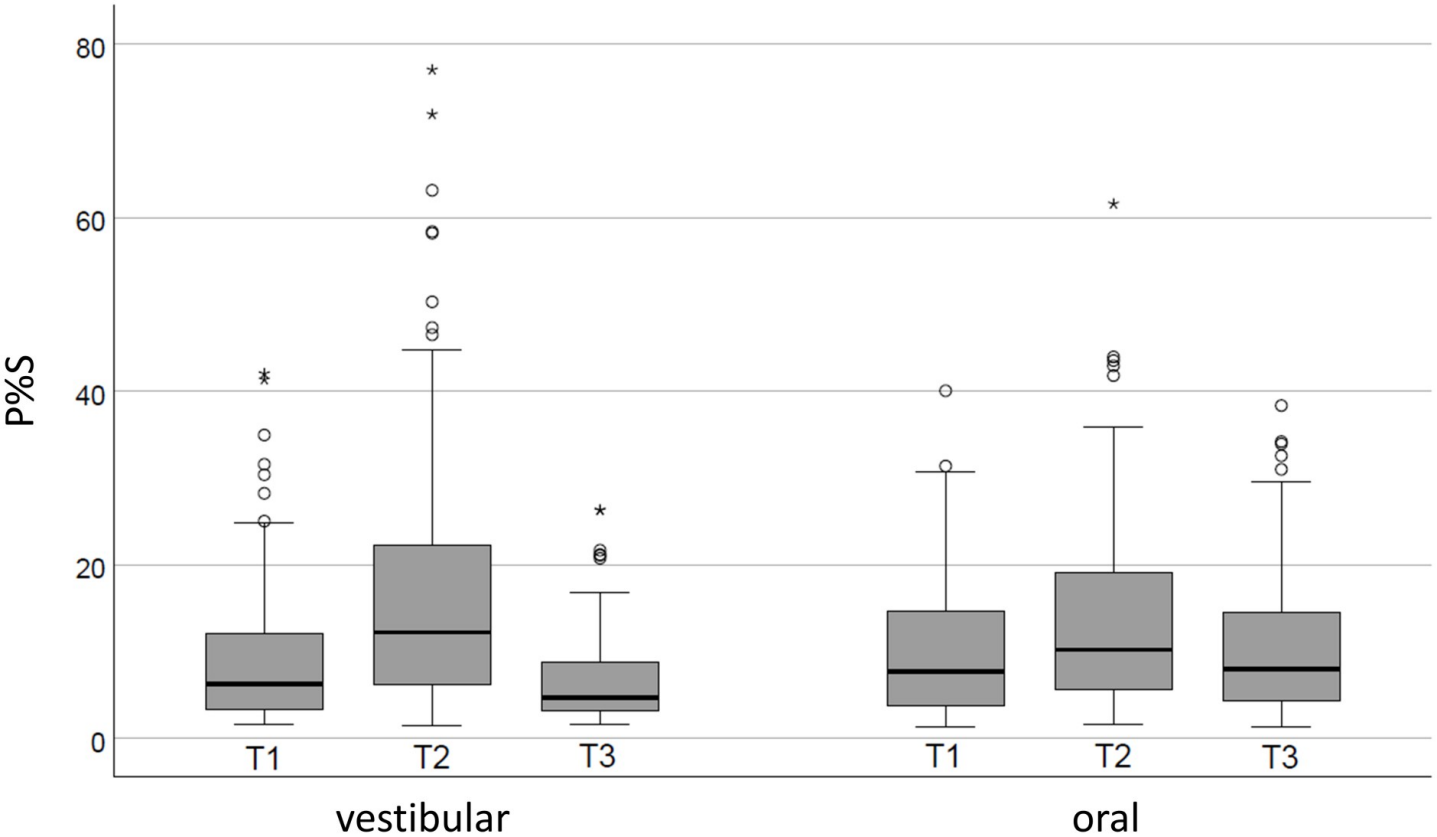
P%S values at T1 (baseline), T2 (after 48 h abstaining from oral hygiene) and T3 (after tooth brushing) are shown for the oral and vestibular surfaces of the Ramfjord teeth. Boxplots showing the minimum value, first quartile, median, third quartile, and maximum value. Circles indicate outliers (values more than 1.5⋅IQR above the upper quartile (Q3) or below the lower quartile (Q1)), asterisks indicate extreme values (values more than 2.5⋅IQR above the upper quartile (Q3) or below the lower quartile (Q1)).

Plaque levels at the three time points are shown in Fig 5.

On the vestibular surfaces, the median P%S was 6.9 (6.4;11.6) at baseline, increasing to 16.0 (12.1;21.0) at T2 (p≤0.001 compared to T1) and decreasing to 5.2 (4.4;9.4) at T3 (p≤0.001 compared to T2). The shifts in plaque amounts were less pronounced on the oral surfaces, but significant changes were found at all time points. At baseline, the P%S value was 9.4 (7.6;11.6), this increased to 12.0 (10.6;15.3) at T2 (p = 0.004 compared to T2) and fell slightly but significantly to 11.8 (7.2;13.4) after tooth brushing (p = 0.002 compared to T2).

### Analysis at the site level

Table 1. Shows the plaque values overall and for all teeth individually, separated by oral and vestibular surfaces.

**Table 1:** Overall and site-specific P%S values (median (95% confidence interval/min;max)) obtained from images from the 3D-intraoral-scan at T1 (baseline), T2 (after 72 h without oral hygiene) and T3 (after toothbrushing).

	T1	T2	T3
**All**			
	7.0 (8.8;10.8/1.4;42.5)	10.3 (13.7;17.1/1.4;77.1) ●	5.9 (6.2;9.6/1.35;38.4) ♦
**Vestibular**			
**First Incisors**			
Upper left	2.6 (2.3;4.2/1.7;10.7)	3.4 (2.7;6.7/1.4;18.1)	2.2 (2.1;2.7/1.7;7.7)
Lower right	3.4 (3.1;4.6/2.4;10.4)	6.9 (4.3;13.4/2.8:24.7) ●	4.2 (3.4;5.6/2.3;9.2)
**First Premolars**			
Upper left	7.8 (5.1;12.8/2.6;30.4)	15.6 (9.2;23.5/5.3;42.6) ●	4.8 (3.8;7.6/2.7;15.4) ♦
Lower right	6.0 (5.0;10.6/2.7;22.6)	10.1 (6.7;12.2/3.4;46.5)	5.5 (4.0;10.3/3.0;16.3) ♦
**First Molars**			
Upper right	12.2 (7.1;17.0/2.5;42.1)	38.6 (27.5;47.6/4.1;77.1) ●	6.2 (3.9;11.4/3.0;26.4) ♦
Lower left	10.8 (7.9;14.7/4.1;35.0)	17.2 (12.7;22.6/7.0;35.9) ●	7.7 (5.2;12.7/2.4;21.7) ♦
**Oral**			
**First Incisors**			
Upper left	3.6 (2.4;7.4/1.5;31.4)	5.3 (3.3;6.6/1.7;11.1)	3.8 (2.6;5.3/1.7;12.3)
Lower right	3.9 (2.7;7.4/1.4;25.2)	8.2 (5.9;14.2/1.9;34.8) ●	5.9 (3.6;7.7/1.9;23.2)
**First Premolars**			
Upper left	6.2 (4.6;8.5/3.1;22.3)	7.0 (5.3;11.0/2.7;43.0)	7.4 (5.3;8.6/1.8;21.2)
Lower right	14.7 (9.9;18.2/3.7;40.1)	26.9 (18.7;29.6/5.7;61.6)	12.7 (10.2;23.7/2.7;38.4) ♦
**First Molars**			
Upper right	14.4 (10.1;18.1/5.7;24.8)	15.0 (10.5;19.3/3.3;25.8)	17.2 (10.5;18.8/2.0;21.8)
Lower left	9.5 (6.0;12.4/2.6;29.9)	12.8 (5.6;18.9/2.5;44.0)	10.2 (5.7;17.4/1.4;34.0)

Level of significance = ≤ 0.002 (Bonferroni correction).

^●^ = significant difference between T1 and T2.

^♦^ = significant difference between T2 and T3.

Baseline plaque levels varied between areas: for the vestibular surfaces, the upper and lower molars showed the highest values and the incisors showed the lowest. On oral surfaces, the lower premolars and upper molars showed the highest values and again the incisors the lowest values. Overall, after professional tooth cleaning and subsequent refraining from oral hygiene, the amount of plaque increased by 47% (p≤0.001), and decreased by 43% (p≤0.001) after tooth brushing. When looking at the oral and vestibular surfaces separately, the greatest changes were found on the vestibular surfaces, while the oral surfaces showed almost no significant changes for the amount of plaque at both T2 and T3.

## Discussion

**Fig 6:**
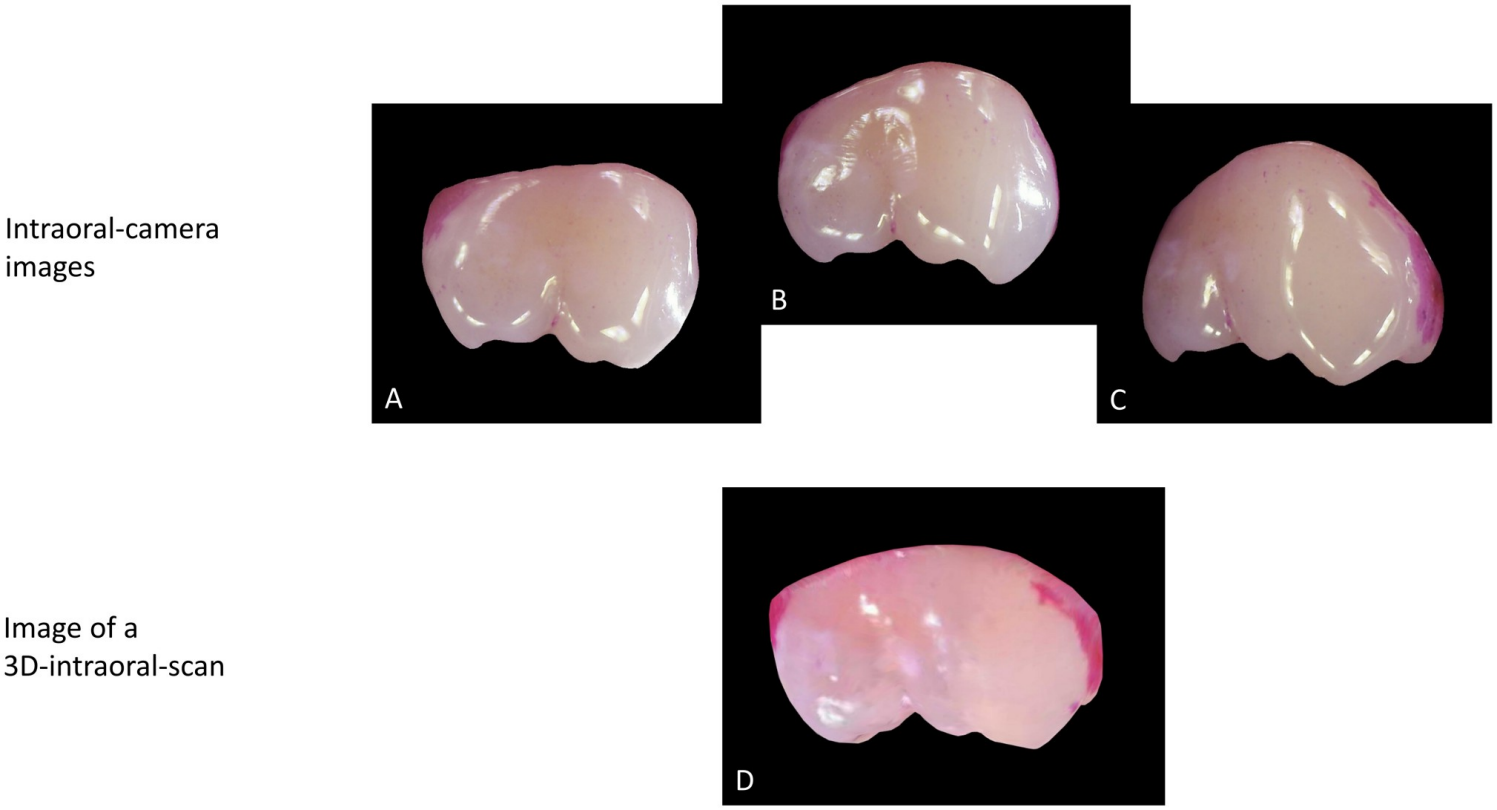
The difference between an intraoral-camera image and the image of a 3D-intraoral-scan of tooth 16 is shown here. While in the intraoral-camera image the proximal surfaces remain hidden in the central image due to the convexity of the tooth, the tooth appears flattened on the image of the 3D-intraoral-scan and the proximal surfaces are more visible. A-C: Intraoral-camera images: A: mesial view, B: central view, C: distal view. D: an image from the 3D-intraoral-scan with a central view.

The aim of the study was to investigate whether planimetric plaque measurement can be validly transferred to images of 3D-intraoral-scans. We used a partial mouth approach focusing on the Ramfjord teeth [24], which are considered sufficiently representative of the entire dentition [25, 26]. As a reference method, we used intraoral-camera images and focused on the vestibular surfaces of the Ramfjord molars. These are relatively straightforward to image in good quality and show the greatest changes in plaque quantity when oral hygiene is suspended and thus the greatest overall range of values. Both aspects are particularly relevant for a validation in the sense of a proof-of-principle. However, a limitation of taking intraoral- camera images is that a tooth surface, especially in the proximal region, it is not fully represented by a single central image due to its convexity. On the images from the 3D-intraoral-scan, this was to a much lesser extent the case as shown in Fig 6.

Accordingly, the P%S values were generally somewhat larger than the P%C values, which was shown to be a systematic bias in the Bland-Altmann analysis. This effect was independent of the magnitude of the plaque quantity. Furthermore, photographs are often not evenly bright, even though we minimised the problem by using an intraoral-camera that does not require a flash. However, the uneven brightness of images makes it difficult to define pixels as plaque-covered and non-plaque-covered. Therefore, photographs, whether taken with an intraoral or extraoral camera, have limitations as a reference method.

Our extensive preliminary work has shown that the tilt and rotation of the 3D-intraoral-scans have a significant effect on the appearance of a single tooth surface and, consequently, on the percentage of the surface covered with plaque. This problem probably also applies to camera images. It is very difficult to capture intraoral images of the molars as well as the oral surfaces in a uniformly centered and completely reproducible manner. Limiting factors here are the cheeks and tongue, which make it difficult to maintain equal angles and distances. However, this is much easier to achieve with intraoral-cameras than with images taken with conventional cameras using mirrors. Until now, this has meant that planimetric examinations have concentrated mainly on anterior teeth [6, 27, 28], since standardized settings are easier to achieve here than in the posterior region due to the use of head and camera mounts.

The major advantage of 3D-intraoral-scans in this context is that soft tissue and other anatomical features are not visualized, and the 3D-intraoral-scan can be freely rotated and adjusted on the screen. This makes it possible to define alignments for all tooth surfaces and all teeth, whether anterior or posterior, and apply them equally to all 3D-intraoral-scans.

In order to bring the alignment of the scan for all examinations points as close as possible into agreement, we first aligned the baseline intraoral-scan in such a way that the tooth surface in question was represented in the best possible way and then aligned the subsequent intraoral-scans accordingly in a direct comparison. When cropping the tooth surface, we also used the baseline images as a guide, as it was not always possible to clearly delineate the respective oral or vestibular surface in some teeth due to their anatomical structure. This was especially the case with the oral surfaces of the lower premolar. In this tooth type, the cusp is often only slightly prominent and merges into the marginal ridge without any transition. Both of these methodological issues were the reason why the evaluation was not blinded according to examination time. However, since the plaque values were computer-generated after cropping all the tooth surfaces of a subject, this probably had little influence on the results. For the evaluation of the cropped images, a separate programme was developed that allows both batch evaluation with fixed pre-settings but also the individual evaluation with customised settings. The former was well applicable for the intraoral-scan images, which had uniform illumination and brightness as well as colour representation. The intraoral-scan images were therefore evaluated using batch processing. This was not possible for the intraoral-camera images, so the settings for each image were adjusted individually in comparison to the original until the delineation of the plaque-covered area corresponded as well as possible to the visual aspect.

The comparison of the images from the 3D-intraoral-scan with the intraoral-camera images showed some outliers, which are not only due to the different representation of vaulted tooth surfaces. On closer inspection, we were able to attribute much of this to a different colouration of the plaque. Although staining was performed twice, the stain on the oral surfaces in the mandible was washed out relatively quickly due to saliva exposure. This was less the case at T3, because the remaining plaque was stained again in the same session, so that a total of four dyeings were carried out. This resulted in some cases in a more intensive colouring of the remaining plaque and supposedly higher P%S values after brushing. Therefore, much emphasis should generally be put on staining procedures in future plaque planimetry studies.

The plaque quantities we measured at the different points in time correspond very well to the known plaque distribution pattern.

An older study examined the plaque distribution under habitual oral hygiene and showed that on the vestibular surfaces of the upper jaw, the most plaque is found in molars and the least in incisors, while the plaque distribution on the oral surfaces is relatively even. In the lower jaw, the vestibular surfaces of the molars also exhibited the greatest amount of plaque, but the difference to the other tooth groups is less pronounced than in the upper jaw [29]. In the present study, these patterns are consistent with the study mentioned above, with the exception of the anterior teeth, which showed only low plaque coverage overall. This was, however, similar to what we found in our earlier study [8].

The pattern of plaque growth after 72 hours without oral hygiene conform to the findings of an earlier study showing that plaque growth on the vestibular surfaces was much more pronounced in premolars than in incisors (molar and oral surfaces were not examined) [30]. The present results are also in very good agreement with those of our previous study, in which we found the major changes in plaque amount on the vestibular surfaces after 72 hours without oral hygiene, while the oral surfaces showed little change [8]. In both studies, plaque growth was most pronounced on the buccal surfaces of the maxillary posterior teeth.

As expected, plaque levels decreased after brushing, but mainly on vestibular surfaces; oral surfaces showed almost unchanged plaque levels. This was also the result of our previous study [8]. In general, video-observation studies show uniform behavioural patterns when brushing their teeth [31–33]. The vestibular surfaces are brushed significantly longer than the oral surfaces and many subjects do not reach the oral surfaces at all despite a long total brushing time. Even though we did not observe toothbrushing in the present study, the low level of plaque reduction on the oral surfaces could be due to this widespread brushing pattern.

A limitation of our study is that we did not include subjects with periodontal disease or metallic restorations; both can significantly affect the quality of the scans. Further studies must show to what extent 3D-intraoral-scanner are also suitable for these situations.

## Conclusions

This study shows that planimetry on 3D-intraoral-scans appears to be a valid and promising tool for plaque detection and monitoring.

Due to the homogeneous illumination of the 3D-intraoral-scan, batch processing is possible in contrast to intraoral-camera images, which makes the evaluation very fast and effective. With the same image quality, the intraoral-scan eliminates the anatomical factors that impede or prevent a central view of the tooth under examination compared to intraoral-camera images, thus enabling a consistently good planimetric evaluation of all tooth surfaces throughout the whole dentition.

All authors have approved the final version to be published and agreed to be accountable for all aspects of the work in ensuring that questions related to the accuracy or integrity of any part of the work are appropriately investigated and resolved.

## Supporting information

S1 File (XLSX)

S2 File (XLSX)
